# Moebius syndrome—Case report

**DOI:** 10.1002/ccr3.6715

**Published:** 2022-12-13

**Authors:** Dan Cristian Gheorghe, Adina E. Stanciu, Adina Zamfir‐Chiru‐Anton, Oprea Doru, Veronica Epure

**Affiliations:** ^1^ Carol Davila University of Medicine and Pharmacy Bucharest Romania; ^2^ ENT Department “MS Curie” Hospital Bucharest Romania; ^3^ Institute of Oncology Bucharest Department of Carcinogenesis and Molecular Biology Bucharest Romania; ^4^ "Grigore Alexandrescu" Children's Emergency Hospital ENT Department Bucharest Romania

**Keywords:** abducens nerve palsy, congenital facial palsy, Moebius syndrome

## Abstract

Moebius Syndrome is a rare multifactorial condition defined by congenital complete or partial VII and VIth cranial nerves palsy and other physical abnormalities. We present the case of a 3 months old infant with Moebius sequence and breathing and eating difficulties, managed by tracheostomy and laryngoplasty.

## Graphical abstract

Moebius Syndrome is a rare congenital condition defined by cranial nerves palsy and other physical abnormalities. Eating and breathing difficulties can occur in these babies and can by successfully managed by tracheostomy

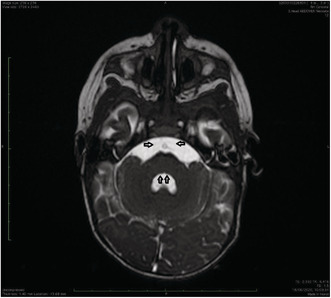

## 1 INTRODUCTION

Moebius syndrome (MBS) or Moebius sequence^1^ is a rare, nonprogressive, neurological congenital defect characterized by uni‐ or bilateral congenital 7th and 6th cranial nerve paralysis and less commonly abnormalities of other cranial nerves (CN) III, IV and IX‐XII, craniofacial, odontological, ophthalmological and orthopedic anomalies.

There are other genetic syndromes that affect the cranial nerves functions and limbs morphology, including Pierre‐Robin sequence, Poland sequence, Carey‐Fineman‐Ziter Syndrome and hypoglossal‐hypodactyly syndrome.^1^ Facial diplegia, gaze palsy, dysphagia and breathing difficulties are associated with MBS. Approximately 90% of the newborns present with airway obstruction from associated craniofacial anomalies.^2^ Signs and symptoms may include microstomia, glossoptosis, high‐arched palate, micrognathia, retrognathia, cleft palate, drooling, nasopharyngeal reflux, gastroesophageal reflux, and laryngomalacia. A vast array of limb deformities—brachy and/or syndactily, Talipes equinovarus, have been described.

The reported incidence of MBS is 1 case per 250,000 live births, with no sex variation. Prognosis is strictly dependent on the degree of patient's deficits but, with early recognition of the disease, there is hope for average developmental results by the age of 5.^3^

Management of MBS patients remains supportive and symptomatic, with main focus on restoring normal breathing and feeding functions when needed and long term psychological, physical, occupational and speech therapy.

We present the case of an infant with MBS who presented to our hospital with breathing and feeding difficulties and discuss the challenges around the diagnosis and treatment of such a patient. Written informed consent from the child's parents and approval from the Hospital's Ethics Board were obtained.

## 2 CASE REPORT

A 3‐month‐old male infant presented to our department for persistent breathing and feeding difficulties. The patient was born during the total lockdown of COVID‐19 pandemic, a full‐term born neonate from a multiparous woman with no history of maternal drug intake. Patient's siblings had no past medical problems. During pregnancy, the mother developed polyhydramnios, with normal FISH Test from the amniotic fluid. Delivery was by C‐section, due to Nuchal cord, with precocious Neopuff at birth and difficult transition to extrauterine life, being admitted into neonatal intensive care unit (NICU).

On admission to the NICU the patient underwent a cytogenetic blood test with no noticeable chromosomal anomalies but a normal male karyotype. He also had feeding difficulties, with no swallow reflex and feeble suckling reflex, necessitating nasogastric tube feeding, normal hearing test (bilateral otoacoustic emission test—PASS) and intermittent laryngeal stridor. Due to the COVID‐19 pandemic restrictions the patient did not undergo an ENT endoscopic evaluation at first. Later ENT evaluation showed inferior ankyloglossia, with surgical correction being performed in the same checkup. A suspicion of Pierre Robin sequence was raised.

**FIGURE 1:**
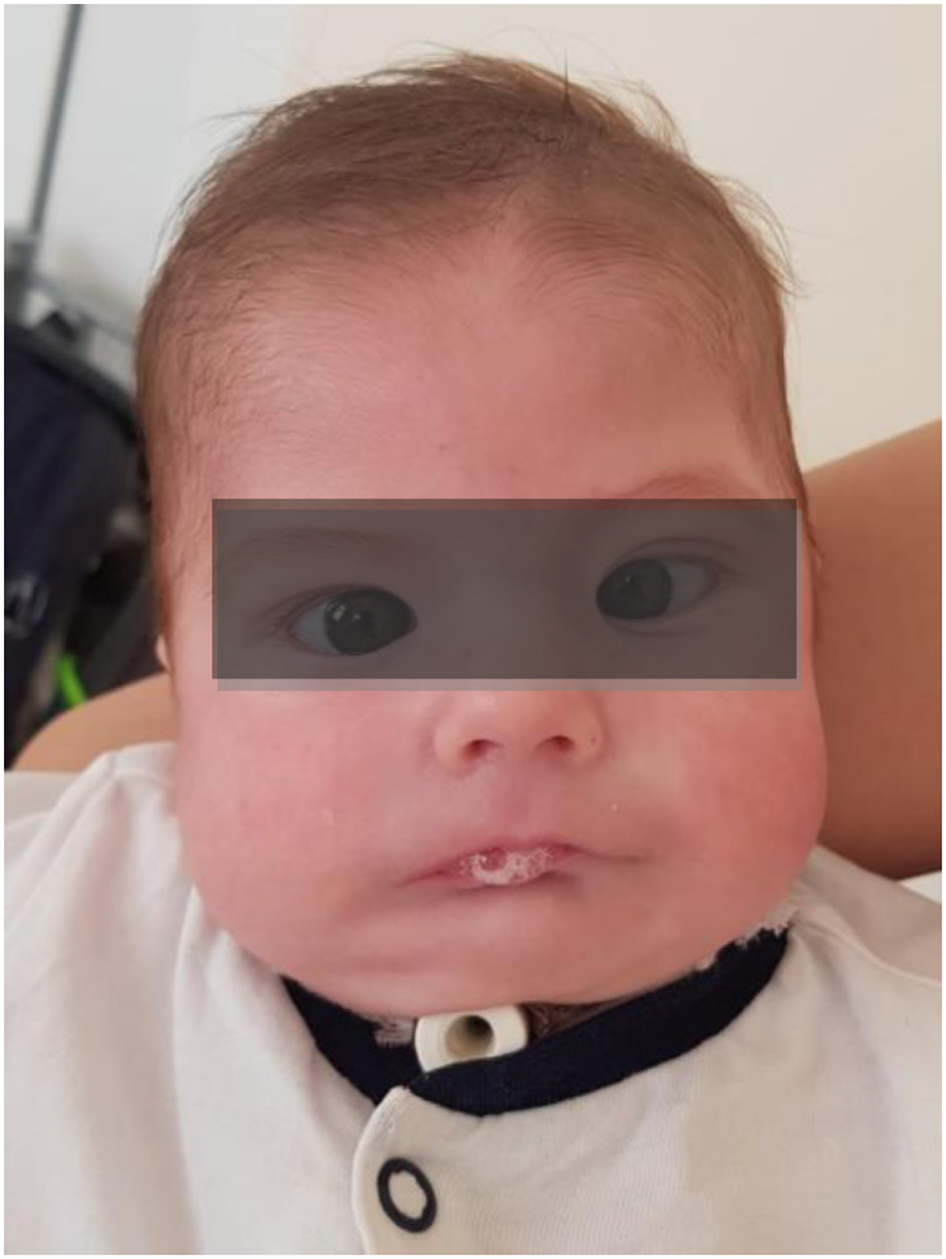
Mask‐like face appearance of MBS patient, with wide nasal root, oblique palpebral fissure, bilateral convergent strabismus (gaze palsy) and ptyalism.

**FIGURE 2:**
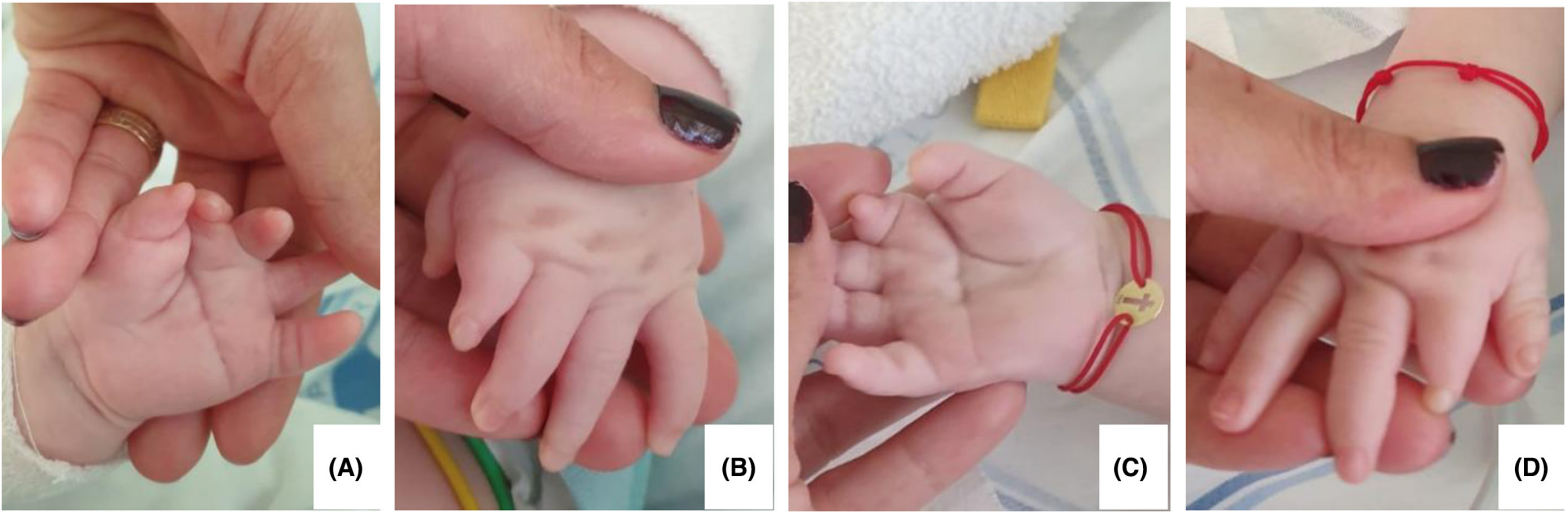
(A) Left hand brachydactyly and simian crease; (B) left hand brachydactyly; (C) right hand brachydactyly and simian crease; (D) right hand brachydactyly.

Finally, at admission in our Department, the patient had a poor general status, with marked restlessness, chronic respiratory distress with nasal flaring, intercostal and suprasternal retractions and inspiratory stridor. Feeding was performed through a nasogastric tube. Other findings included: lack of facial expression, inability to fully close the eyes, convergent bilateral strabismus, drooling, dysmorphic facial features—oblique palpebral fissure, wide nasal root, abnormal nose shape, mandibular hypoplasia, a short neck, bilateral brachydactyly and bilateral simian crease (Figures 1 and 2). Apart from a high‐arched palate, there were no other findings of anatomic facial abnormalities.

We performed a flexible awake fiber optic laryngoscopy that revealed laryngomalacia: short aryepiglottic folds and large arytenoids obscuring glottis view in inspiratory phase, normal movement of vocal folds and no abnormalities of the subglottic and tracheal airway.

**FIGURE 3:**
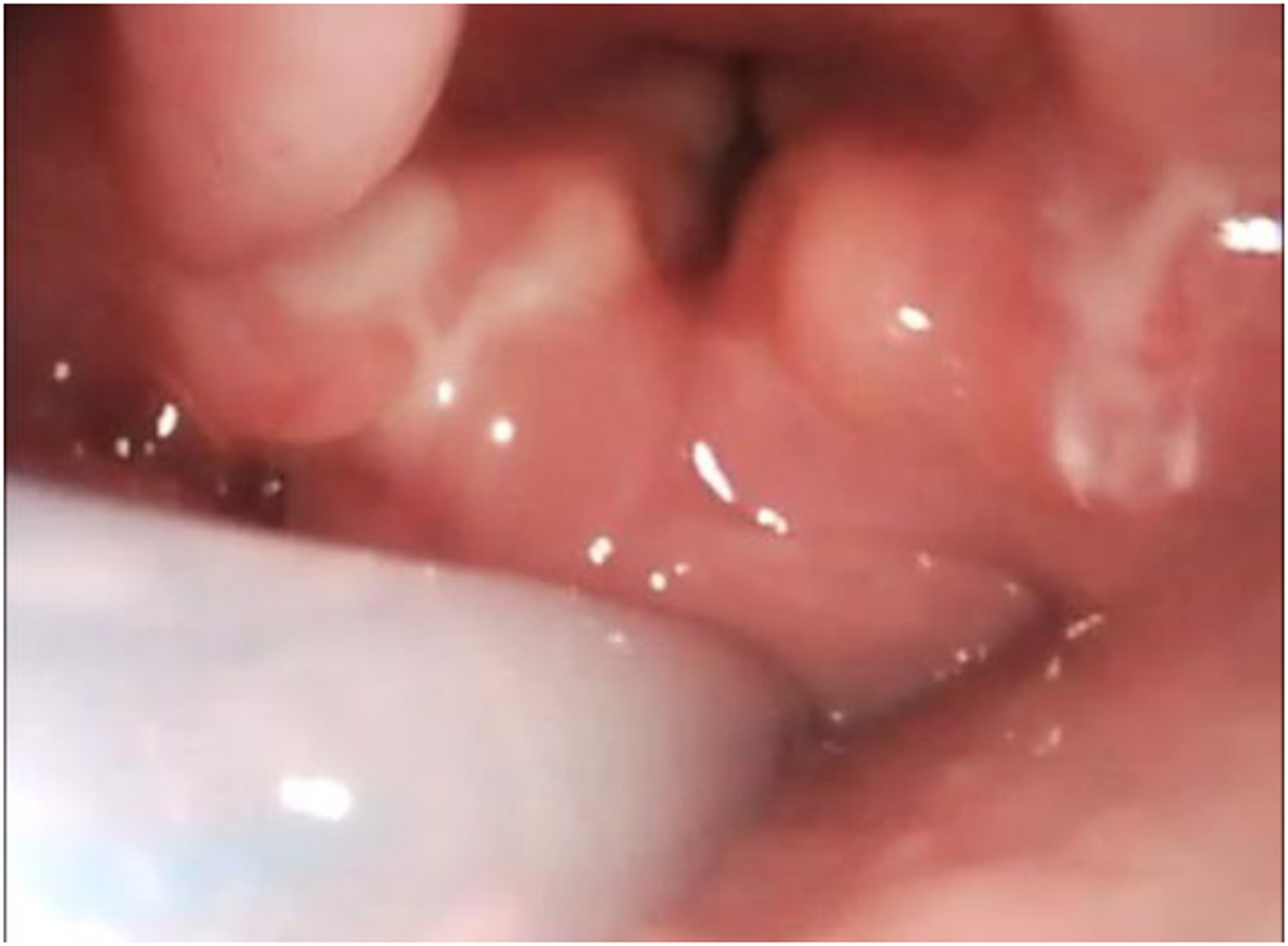
Fibroscopic view of larynx during expiratory phase after supraglottoplasty.

Performing a temporary tracheostomy was decided based on aspiration risks suggested by the obvious neurological feeding difficulties and also in contemplating the need of a supraglottoplasty in order to relieve respiratory distress. Suspension microlaryngoscopy was performed with unilateral supraglottoplasty, reducing the volume of the left arytenoid mucosal layer and incising both aryepiglottic folds with cold instruments. Three days later we performed arytenoid mucosa removal to the opposite side, in order to avoid possible risks of stenosis of the supraglottic structures (Figure 3).

**FIGURE 4:**
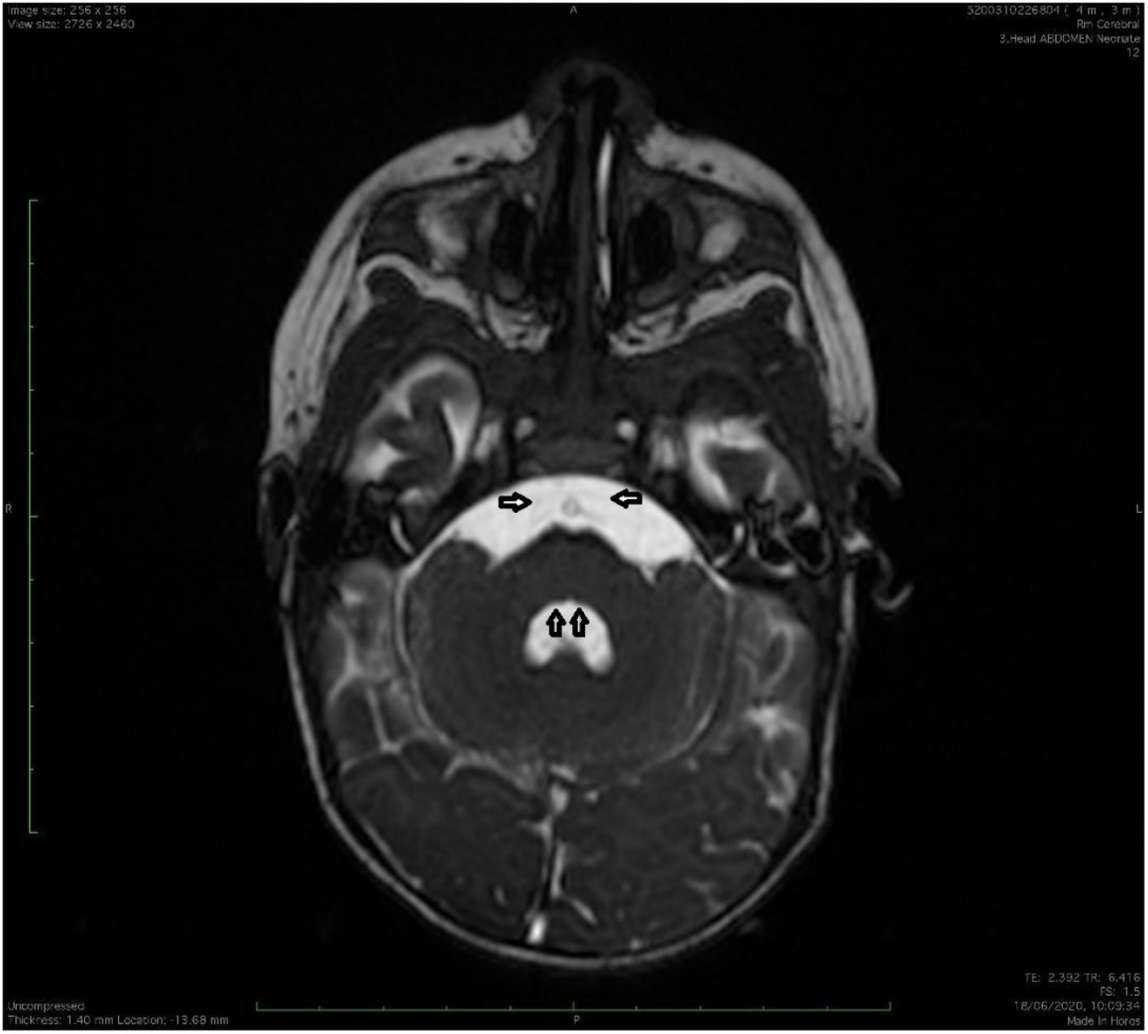
Axial T2‐weighted image shows bilateral absence of the facial colliculus in the pontine tegmentum (vertical arrows). The fourth ventricle has an inverted V shape. At the level of middle cerebellar peduncles there are no radiologic traces of abducens nerves; the arrows (horizontal arrows) depict the normal trajectory of abducens nerves at this level.

The patient underwent an MRI scan revealing the pons with absent medial colliculus and absent abducens nerves (bilaterally), with hypoplastic VIIth nerve fibers and nuclei, suggestive for MBS brainstem alterations (Figure 4).

Seven days after tracheostomy was performed, the tracheal cannula was removed under close monitoring; feeding degraded abruptly during the same day, so the patient had to return to nasogastric tube feeding once again. Throughout the night, the infant had been in full respiratory distress so we had to recannulate the patient, followed by significant general and respiratory status improvement. Interestingly, feeding ability also returned, sleeping habits also improved, allowing the patient to be discharged in good general status and with normal feeding routine.

## 3 DISCUSSION

Moebius Syndrome is a rare and complex condition comprising usually two or more CNs afflictions, typically the 7th and 6th, possibly associated with other limbs or craniofacial malformations and long term consequences on a newborn somatic and cognitive development. Its etiology is either genetic, as several studies point towards each of two 3q21‐q22 or 10q loci, or environmental.

Viewing the complex course and functions of the facial nerve,^4^ the main clinical features of the disease in babies include incomplete closure of the eyelids during sleep, incomplete closure of the lips, mask‐like face appearance (absence of any smiles), poor or absent sucking, breathing difficulties, convergent strabismus, occasionally associated with limb abnormalities like brachydactyly, syndactyly or Talipes equinovarus.^2^

It is worth mentioning that although mandibular hypoplasia was noticed, no other Pierre Robin sequence anomalies were present. The respiratory difficulties were identified as laryngomalacia and managed by surgery (aryepiglottoplasty). We did not properly assess the global muscular function in such a small child but normal muscle development and lack of cleft palate ruled out the diagnosis of Carey Fineman‐Ziter Syndrome.^1^

Even though MBS patients frequently undergo surgical procedures addressing bone deformities, breathing or feeding difficulties or ophthalmological dysfunction, no curative therapeutic resources exist, so early recognition and intervention could improve the outcome on patient's life expectancy and quality of life. In selected cases, a smile procedure can be performed, either by nerve transfer technique, which uses the masseteric nerve to restore innervation in the free gracilis, or by muscle transfer technique (lengthening of the temporalis muscle and suturing it to selected points on the ipsilateral corner of the mouth).^5^

Even though respiratory failure is uncommon in MBS newborn,^6^ different levels of respiratory distress as a consequence of the disease have been described in such patients.

Difficulties were encountered to properly recognize the disease in our presented case. Although the patient had an ENT, pediatric and neurologic examinations, all failed to suspect the presence of the syndrome.

Interestingly, after restoring the child's normal breathing function, which has been our main goal, by tracheostomy and surgical management of laryngomalacia, it proved that the feeding had significantly improved, not only qualitative but also quantitative, removing the need for a nasogastric tube. Thus, there can be little to no feeding problems in MBS newborns.^6^

Other findings like the convergent strabismus, the bilateral brachydactyly and the incomplete bilateral facial nerve palsy are to be addressed individually at later stages of child development, after assuring the patient follows a normal pediatric growth chart.

In our case, due to the small age of the infant, speech, cognition and coordination tests could not be performed, but precocious supportive surgical interventions could prove beneficial in his future, as some studies show that MBS patients grow to have good speaking and reading abilities, and normal intelligence, with only 10%–15% to show some degree of mental retardation.

Decannulation can only be considered at a later stage, after feeding and weight development record a normal pattern, according to patient's age. Permanent tracheostomy can be taken into consideration if no additional surgery of the upper airways can prove beneficial.

Surgery is only symptomatic, but restoring of facial movement should best be addressed before the age of 4, before the patient reaches school age, with contribution to both practical and psychosocial behavior.

## 4 CONCLUSION

MBS is a rare disease with heterogeneous diagnostic criteria leading to an increased chance of misdiagnosis. Investigation of such patients should include mainly an MRI of the brainstem.

Amongst the associated symptoms, respiratory distress and feeding difficulties due to craniofacial anomalies are common findings. These patients should be referred to the ENT specialist, besides detailed examination by the pediatrician, ophthalmologist, neurologist, psychotherapy and support groups for the parents.

## AUTHOR CONTRIBUTIONS

**Dan Cristian Gheorghe:** Conceptualization; project administration; validation; writing – original draft; writing – review and editing. **Adina Elena Stanciu:** Data curation; writing – original draft; writing – review and editing. **Adina Zamfir‐Chiru‐Anton:** Data curation; writing – original draft; writing – review and editing. **Doru Oprea:** Data curation; writing – original draft; writing – review and editing. **Veronica Epure:** Supervision; validation; writing – original draft; writing – review and editing.

## CONFLICT OF INTEREST

None

## CONSENT

Written informed consent was obtained from the patient's parents to publish this report in accordance with the journal's patient consent policy.

## Data Availability

Data available on request due to privacy/ethical restrictions

## References

[1] Pedersen LK , Maimburg RD , Hertz JM , et al. Moebius sequence ‐a multidisciplinary clinical approach. Orphanet J Rare Dis. 2017;12(1):4. doi:10.1186/s13023-016-0559-z 28061881 PMC5217236

[2] Cuestas G , Quiroga V , Zanetta A , Giménez E . Manejo de la vía aérea en el neonato con síndrome de Moebius. An Pediatr (Barc). 2019;91:264‐267. 10.1016/j.anpedi.2018.11.008 30583993

[3] Picciolini O , Porro M , Cattaneo E , et al. Moebius syndrome: clinical features, diagnosis, management and early intervention. Ital J Pediatr. 2016;42(1):56. doi:10.1186/s13052-016-0256-5 27260152 PMC4893276

[4] Poutoglidis A , Paraskevas GK , Lazaridis N , et al. Extratemporal facial nerve branching patterns: systematic review of 1497 cases. J Laryngol Otol. 2022;136(12):1171‐1176. 10.1017/S0022215121003571 36017719

[5] Choi JY , Kim HJ , Moon SY . Management of the paralyzed face using temporalis tendon transfer via intraoral and transcutaneous approach: temporalis tendon transfer. Maxillofac Plast Reconstr Surg. 2018;40(1):24. doi:10.1186/s40902-018-0160-6 30206539 PMC6123326

[6] Sjögreen L , Andersson‐Norinder J , Jacobsson C . Development of speech, feeding, eating, and facial expression in Möbius sequence. Int J Pediatr Otorhinolaryngol. 2001;60(3):197‐204. doi:10.1016/s0165-5876(01)00532-8 11551610

